# DAMIAN: an open source bioinformatics tool for fast, systematic and cohort based analysis of microorganisms in diagnostic samples

**DOI:** 10.1038/s41598-019-52881-4

**Published:** 2019-11-14

**Authors:** Malik Alawi, Lia Burkhardt, Daniela Indenbirken, Kerstin Reumann, Maximilian Christopeit, Nicolaus Kröger, Marc Lütgehetmann, Martin Aepfelbacher, Nicole Fischer, Adam Grundhoff

**Affiliations:** 1 Heinrich-Pette-Institute (HPI), Leibniz Institute for Experimental Virology, Research Group Virus Genomics, Hamburg, Germany; 2 Bioinformatics Core, University Medical Center Hamburg-Eppendorf, Hamburg, Germany; 3 Department of Stem Cell Transplantation, University Medical Center Hamburg-Eppendorf (UKE), Hamburg, Germany; 4 Institute of Medical Microbiology, Virology and Hygiene, University Medical Center Hamburg-Eppendorf (UKE), Hamburg, Germany; 5 German Center for Infection Research, DZIF, partner site Hamburg-Borstel-Lübeck-Riems, Germany

**Keywords:** High-throughput screening, Infectious-disease diagnostics, Molecular medicine

## Abstract

We describe DAMIAN, an open source bioinformatics tool designed for the identification of pathogenic microorganisms in diagnostic samples. By using authentic clinical samples and comparing our results to those from established analysis pipelines as well as conventional diagnostics, we demonstrate that DAMIAN rapidly identifies pathogens in different diagnostic entities, and accurately classifies viral agents down to the strain level. We furthermore show that DAMIAN is able to assemble full-length viral genomes even in samples co-infected with multiple virus strains, an ability which is of considerable advantage for the investigation of outbreak scenarios. While DAMIAN, similar to other pipelines, analyzes single samples to perform classification of sequences according to their likely taxonomic origin, it also includes a tool for cohort-based analysis. This tool uses cross-sample comparisons to identify sequence signatures that are frequently present in a sample group of interest (e.g., a disease-associated cohort), but occur less frequently in control cohorts. As this approach does not require homology searches in databases, it principally allows the identification of not only known, but also completely novel pathogens. Using samples from a meningitis outbreak, we demonstrate the feasibility of this approach in identifying enterovirus as the causative agent.

## Introduction

Nucleic acid based detection of pathogens has widely replaced culture based laboratory methods for the identification of putative pathogens in samples from patients with infectious diseases^1,2^. These procedures are commonly amplification-based and biased because they require a correct hypothesis with regard to the specific infectious agents involved in an infectious disease. Less biased approaches interrogate highly conserved regions (e.g. 16S rRNA bacteria and ITS sequences for fungi) or employ amplification protocols with pan-primer mixes for individual viral families^3–5^. Alternatively, multiplex PCR approaches with multiple primer sets and detection probes in a single tube may be used for specific infectious syndromes (e.g. encephalitis, acute gastroenteritis, pneumonia or severe respiratory distress syndrome). Still, a priori knowledge of specific pathogen is necessary and very often these methods, although highly sensitive, remain negative.

Unbiased next-generation sequencing (NGS) of diagnostic samples is now widely considered a key technology that will fundamentally improve infectious disease diagnostics^2,6–9^. Due to the principal potential to identify not only known but also novel pathogens, such methods are also expected to strengthen the level of preparedness for future outbreaks of emerging pathogens^10^. Decreasing reagent cost and availability of affordable bench top sequencing instruments with relatively low infrastructure demands have promoted the establishment of next-generation sequencing platforms in many hospitals or microbiology laboratories and make this technique highly attractive to improve pathogen detection in diagnostics^11–13^. However, there is still a lack in open source bioinformatic tools that are specifically designed for clinical settings.

Here we describe a user-friendly open source software, which enables clinical personnel without a background in bioinformatics to accurately, and rapidly identify potentially pathogenic agents in clinical specimen. Notably, DAMIAN (Detection & Analysis of viral and Microbial Infectious Agents by NGS) goes beyond taxonomic classification of sequence reads. Its capabilities include functional sequence analysis, which allows for reliable results even in the case of truly novel emerging pathogens not represented in sequence databases. Furthermore, the ability to process cohorts make it a valuable tool for the analysis of outbreak samples. To the best of our knowledge, this is the first software for the detection of pathogens to provide such features. Here, we demonstrate that DAMIAN achieves excellent detection capabilities and an unprecedented level of guidance in the interpretation of analysis results.

## Results

### Description of DAMIAN features and data processing steps

DAMIAN provides capabilities to rapidly identify known and novel infectious agents in samples of various sources. It integrates all required processing steps, ranging from the quality control of raw reads to the generation of comprehensive reports, into a single user-friendly software system. Being intended for the employment in clinical diagnostics, DAMIAN does require neither specialized computational infrastructure nor expertise in bioinformatics to accomplish its tasks. It works for both DNA and RNA samples and, if desired, takes into account almost any host organism to subtract background reads.

Many taxonomic classification tools (e.g. Taxonomer, SURPI or Kraken^11,14,15^) aim at taxonomically classifying single reads. Such an approach is able to deliver results quickly and, at least in cases where adequate reference sequences are available also allows for a solid classification. By contrast, DAMIAN pursues a different strategy and assembles reads into longer contigs prior to classification and annotation. The longer sequences increase the sensitivity and specificity of sequence similarity searches and therewith the quality of taxonomic assignments. Moreover, they allow for a functional annotation, which provides valuable information even when sequence similarity searches do not yield significant matches, and permit cross-comparison of sequence contig signatures across multiple sample cohorts.

**Figure 1:**
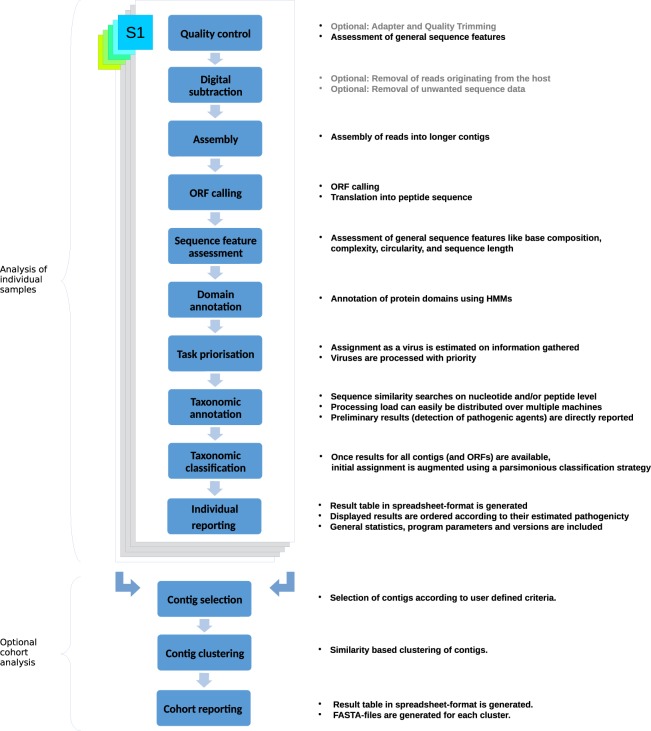
Schematic representation of the data processing steps performed by DAMIAN. Depicted are the individual modules in the DAMIAN workflow starting with FASTQ as input files for the analysis. Individual samples are generally processed independently first. An optional cohort analysis can later be performed on any number of previously processed samples.

The minimum requirement for starting an analysis with DAMIAN are reads in (gzip-compressed or uncompressed) FASTQ-format. Any number and combination of paired-end reads and single-end reads is supported. In the following, we briefly describe the features and processing steps in their actual order of execution during analysis with DAMIAN (Fig. 1).

Upon starting, DAMIAN performs checks to ensure that all requirements for successfully conducting an analysis are met. User input, database connectivity, file permissions and software dependencies are validated and at the same time information, like software versions and parameters given on the command line, are aggregated and stored.

#### Quality control and self-documentation

DAMIAN automatically removes low quality bases and sequencing adapter sequences. Prior processing with external tools is not required. DAMIAN automatically documents every single analysis step and provides gathered information in its analysis results. This information comprehensively describes an analysis and allows for exact reproduction. During the quality control step, for example, information is collected on how quality and adapter trimming effects read properties. Although not explicitly mentioned in the following paragraphs, a similar behavior was implemented for all analysis steps.

#### Digital subtraction and abundance estimation of unwanted sequence reads

In general, sequence reads originating from the host organism are removed and counted. However, this is optional and DAMIAN can be used with any number of different reference sequences or none at all. DAMIAN is able to discriminate between RNA and DNA data and suitable reference sequences can be selected accordingly.

#### Assembly and assessment of basic contig features

Reads remaining after the preceding steps are assembled into larger contigs. Features like length, circularity, GC-content and sequence complexity are determined for each contig and sequences of ORFs are translated into amino acid sequences.

#### Functional annotation and contig ranking

The amino acid sequences are screened for known protein domains. The domains are classified according to the taxonomic entities they are associated with. Some domains, for example, are only found in viruses while others are specific to bacteria or fungi. As the protein domains are functional regions, they can provide unmistakable results even when BLAST searches yield no significant matches with the sequences of known pathogens. Additionally, information on functional domains allows to rank the contigs for subsequent processing such that contigs potentially originating from pathogenic agents are processed first.

#### Taxonomic assignment

DAMIAN employs the complete NCBI nt and nr databases to perform classifications. Searches with nucleotide and derived amino-acid sequences can be performed independently, iteratively or redundantly. Preliminary results are reported whenever a contig yields significant matches to a known microbial or viral agent. In addition to lowest common ancestor (LCA) based taxonomic assignments, DAMIAN also incorporates a two-pass method for taxonomic assignment. It aims at determining which species are present in a sample based on aggregated information from all contigs instead of assigning each contig individually.

#### Reporting

DAMIAN provides a comprehensive report in spreadsheet format for each sample (see *Diagnostic Application* section and Datasets S2–S11 for examples). The main page provides an overview of detected taxonomic entities. Entries are sorted and color-coded to allow for a quick identification of potentially pathogenic agents. The color-code depicts six different categories, red, pink, light blue, dark blue, grey and black. The first category (red) contains entries, which were classified as viruses based on sequence similarity and protein domains. For the second (pink) and third category (light blue) there is only evidence for viral sequences from either sequence similarity or protein domains. Phages are generally listed within a separate category (dark blue). The fifth category is for known artifacts or contaminants (grey), which can be defined by the user and the sixth and final category for everything else, bacteria, fungi and parasites (black). Additionally, DAMIAN enables its users to further investigate sequences, which did not yield significant alignments (see cohort analysis below).

The report is interactive and links entries of the main page to detailed views. These views display detailed data regarding the corresponding contigs, ORFs and protein domains. Additionally, nucleotide and amino acid sequences can be accessed. Other pages of the report contain information on general statistics like number of reads, amount of reads originating from the host and the size sequenced fragments. Program versions and parameters used are also part of the report. It is not necessary to wait for the preceding steps to complete before generating a report. Preliminary reports, integrating all information available so far, can be generated at any time.

#### Cohort analysis

The optional cohort-based analysis allows the identification of sequences which may originate from pathogenic agents shared among groups of samples from individuals showing a given disease phenotype. This analysis does not depend on reference databases, taxonomic assignments or similar prior knowledge. Rather, the user assigns any number of samples to a group of known positives, known negatives or of unclassified samples. For example, all samples that belong to a suspected outbreak can be assigned to the group of positives while samples, which are known to be unrelated to the outbreak, would be assigned to the group of negatives. Finally, samples for which it is unsure whether they are part of the outbreak could be assigned to the group of unclassified samples. The pipeline then performs pairwise BLAST alignment amongst all assembled contigs and sorts them into bins according to their sequence similarity. Within each sequence cluster, a score reflecting the degree to which the cluster is preferentially associated with the positive phenotype is calculated. By sorting the clusters according to their score, the user can easily identify those contigs, which are most likely linked to the phenotype in question, and thus select the most promising candidates that may represent causally related pathogenic agents. Results are reported in spreadsheet format and additionally FASTA files are generated from contig sequences for each cluster. While the results table (see Supplementary Dataset S1 for an example) contains taxonomic assignments for those clusters in which individual contig members could be classified, the clustering itself is completely independent of the success or failure of contig classification. Hence, this approach allows for the identification of completely novel pathogens, provided that they are overrepresented in the positive phenotype group.

### Diagnostic application and comparison with existing software (Taxonomer, PathoScope and metaMix)

To verify the ability of our tool to detect pathogens in diagnostic and putative outbreak settings, we applied DAMIAN to a number of specimens derived from patients suspected to suffer from common community- or hospital acquired infections or in the context of public health emergencies (Table 1). Results were compared to those results obtained via Taxonomer BETA and PathoScope pipelines^11,16,17^ (Tables 2–5). While Taxonomer, PathoScope and DAMIAN each incorporate all analysis steps, metaMix requires the results of sequence similarity searches as an input. The way the pre-processing is performed may immediately impact the results of metaMix. Here we used an IDBA-UD assembly and MEGABLAST results to perform the analysis. IDBA-UD was employed since it is also integrated in DAMIAN, and MEGABLAST was used to allow the analysis to complete within a similar time frame as the other tools. metaMix performance may improve if it is run with different, yet computationally more demanding, pre-processing steps. We included it in the comparison, because like DAMIAN and unlike the other two aforementioned tools, it is able to perform an analysis, which is based on contigs. All specimens were pre-analyzed by state of the art diagnostic tests as part of routine analysis procedures. The routine specimens included two respiratory (bronchoalveolar lavages (BALs) 104 and 3157) and one cerebrospinal fluid samples (CSF 7653), while the public health emergency-related specimens comprised one respiratory (BAL 4505) and three stool samples (1, 9792 and 9790). For all samples, we constructed strand specific RNA-Seq libraries from total nucleic acids extracted in a routine diagnostic environment. Libraries were multiplex sequenced on MiSeq or HiSeq2500 instruments with 2.4 to 3.3 million or ~25 million reads per sample, respectively. In general, DAMIAN reported first results after 10–20 minutes. Pathogenic agents were reported within less than an hour in most cases (Table 1).

**Table 1:** Time frame in which clinically relevant results were obtained by DAMIAN.

sample ID^a^	diagnostic entity	detected pathogen(s)	time^b^
104	bronchoalveolar lavage	Influenza A	73
3157	bronchoalveolar lavage	Influenza A	40
4505	bronchoalveolar lavage	Chlamydophila psittaci	n/a
9790	stool	human Parechovirus	44
9792	stool	Sapporovirus	38
		human Parechovirus	38
1	stool	Norwalk Virus	162
7653	cerebrospinal fluid	Enterovirus B	17
SRR1553464	serum	Zaire Ebolavirus	13
SRR533978	serum	Bas Congovirus	18
SRR1564804	plasma	Chlamydophila psittaci	n/a

^a^Diagnostic sample or public available dataset (SRR1553464, SRR533978, SRR1564804) analyzed by DAMIAN.

^b^Time (in minutes) until the first report of a putative pathogen was received.

n/a: not applicable; time frames are only calculated for viral contigs since DAMIAN prioritizes viral sequences. The analysis was performed using 12 threads of a server with two Intel Xeon E5-2687W v3 CPUs.

**Table 2:** Comparison of BAL sample analysis results obtained by DAMIAN, Taxonomer BETA, PathoScope and metaMix.

	104	3157	4505
DAMIAN	23,828,285 reads 99,23% human sequences	3,265,314 reads 51,74% human sequences	2,370,210 reads 98,13% human sequences
	✓ **Influenza A** (**39**,**810 reads**; **42%**) • H1N1 all 8 segments (8 contigs) 97–100% id. ○ Influenza A ○ A/Singapore/TT198/2011 (H1N1) ○ A/Swine/France/71-130116/2013 (H1N1) ○ A/Swine/France/71-130116/2013 (H1N1) ○ A/Singapore/TT198/2011 (H1N1) ○ A/Santa Clara/YGA_03065/2013(H1N1), ○ A/Arizona/M2/2012(H1N1) ○ A/Swine/France/71-130116/2013 (H1N1) ✓ **Candida albicans (14**,**249 reads**, **15**.**15%)**	✓ **Influenza A (1**,**886 reads**, **1**.**57%)** • H3N2 all 8 segments (8 contigs) 99% id. ○ PB2, A/Connecticut/Flu140/2013(H3N2) ○ PB1, A/Connecticut/Flu140/2013(H3N2) ○ PA, A/Connecticut/Flu140/2013(H3N2) ○ HA, A/Connecticut/Flu140/2013(H3N2) ○ NP, A/Connecticut/Flu140/2013(H3N2) ○ NA, A/Connecticut/Flu140/2013(H3N2) ○ M2, M1, A/Connecticut/Flu140/2013(H3N2) ○ NEP,NS1, A/Connecticut/Flu140/2013(H3N2) ✓ **human parainfluenza 3 virus (1 read**) 99% id. ✓ **human herpes simplex virus 1 (4 reads**) 99% id. ✓ **Candida albicans (10**,**207 reads**, **7**.**65%)**	✓ **Chlamydophila psittaci (237 reads; 4**.**13%)** 100%id. • Chlamydophila psittacci 6BC, 4 contigs, 16S and 23S rRNA • Chamydophila VS225, 1 contig, 16S rRNA • Chlamydophila Mat116, 1 contig, 16S rRNA
Taxonomer BETA	9,900,000 reads sampled^*^; 5% classified Bacteria: 62,128 reads; Viruses: 10,723 reads; Fungi: 86 reads	3,200,000 reads samples; 13% classified Bacteria: 300,309 reads; Viruses: 7,686 reads; Fungi: 17,878 reads	2,300,000 reads samples; 4% classified Bacteria: 24,960 reads; Viruses: 950 reads;
	✓ **Influenza A (1**,**227 reads)** • H1N1 (1,227 reads) ✓ α-retrovirus (9,677 reads) ✓ dsDNA virus (505 reads)	✓ **Influenza A (1**,**433 reads)** • H3N2 (354 reads) ✓ **Human parainfluenza 3 virus (13 reads)** ✓ α-retrovirus (907 reads) ✓ Caudovirales (1,299 reads) ✓ **Herpesviridae** (184 reads) ✓ **Candida albicans (15**,**537 reads)**	✓ **Chlamydia (75 reads)** • **Chlamydia psittaci (33 reads)** • **Chlamydia trachomatis (30 reads)** ✓ Proteobacteria (510 reads) ✓ Firmicutes (33 reads) ✓ α-retrovirus (169 reads) ✓ Herpesviridae (52 reads)
PathoScope	45,576 aligned reads; 2,234 hits ✓ **Influenza A (12**,**234 reads)** • Subtypes H3N2; H5N1; H1N1; H9N2; H2N2 ✓ Hepatitis C (224 reads) • Genotype 2; 1; 6 ✓ Encephalomyocarditis Virus (115 reads)	184,719 aligned reads; 2,434 hits ✓ **Influenza A (1**,**337 reads)** ✓ Subtypes H3N2 ✓ Avian leukosis virus (1,256 reads) ✓ **human herpes simplex virus 1 (102 reads)** ✓ Veillonella parvula (130,363 reads) ✓ Enterococcus faecium (21,178 reads)	4,408 aligned reads; 1,752 hits ✓ **Chlamydophila psittaci (23**.**83 reads)#**
metaMix	26 hits; 7,328,046 human reads ✓ **Influenza A (1 contig; 46**,**698 reads)** • H1N1, 1 contig; A/Canela/LACENRS-418/2013 ✓ **Candida albicans SC5314 2 contigs (601**,**527 reads)** Bacteria 3 contigs (372 reads)	44 hits; 201,764 human reads ✓ **Influenza A (3 contigs; 2**,**244 reads)** • H3N2, 2 contigs; A/Bage/LACENRS-205/2013; A/Porto Alegre/LACENRS-275/2013 ✓ **Candida albicans**, **1 contig (97**,**816reads)** Bacteria; 15 contigs (109,048 reads)	16 hits; 142,965 human reads ✓ **Chlamydia psittaci (1 contig; 252 reads)**

^*^Files >5GB are not supported by taxonomer BETA version; 10,000,000 reads were randomly sampled to meet 5 GB maximum size for upload. *Files >5GB are not supported by taxonomer BETA version; 10,000,000 reads were randomly sampled to meet 5 GB maximum size for upload. # fractional read abundance given by PathoScope.

**Table 3:** Comparison of stool sample analysis results obtained by DAMIAN, Taxonomer, PathoScope and metaMix.

	9790	9792	1
DAMIAN	1,667,291 reads 0,64% human sequences	1,347,375 reads 0,16% human sequences	23,292,070 reads 1,36% human sequences
	✓ **human parechovirus 6 (3 contigs**, **132 reads**, **0**.**1%) 96–97% id**. ✓ Bacteroides ✓ Bifidobacterium	✓ **Sapporovirus (10**,**028 reads**, **14**.**62%)** • **Sapovirus Hu/G1/BE-HPI01/DE/2012** ✓ **human parechovirus 1 (3 contigs; 370 reads**, **0**.**54%) 90–97% id** ✓ Bifidobacterium	✓ **Norwalk Virus (1**,**163**,**565 reads**, **8**.**22%)** •**Primate Norovirus strain simianNoV-nj**, **complete genome 98% id (1 contig**, **757**,**078 reads)#** **•Chiba Virus genomic RNA**, **complete genome 93% id (1 contig**, **402**,**355 reads)** **•Norovirus Hu/GII**.**P16/GII**.**13/New/Taipei/13-BA-1/2013/TW complete genome 99% id (1 contig**, **4**,**132 reads)** ✓ Bacteria
Taxonomer BETA	1,600,000 reads samples; 77% classified Bacteria: 1,207,356 reads; Viruses: 2,493 reads; Fungi: 419 reads	1,300,000 reads samples; 86% classified Bacteria: 925,978 reads; Viruses: 13,005 reads;	9,900,000 reads sampled^*^; 85% classified Bacteria: 8,200,128 reads; Viruses: 100,803 reads; Fungi: 86 reads
	✓ **human parechovirus (89 reads)** • **human parechovirus 6 (10 reads)** • **human parechovirus 1 (7 reads)** ✓ α-retrovirus (1166 reads) ✓ ds DNA viruses (1,334 reads) ✓ ss DNA viruses (353 reads) ✓ Bacteroidetes (492,974 reads) ✓ Actinobacteria (99,321 reads) ✓ Proteobacteria (89,830 reads) ✓ Firmicutes (388,188 reads)	✓ **Caliciviridae (7**,**913 reads)** • **Sapporovirus (562 reads)** **GI (80 reads); GI**.**2 (53 reads)** ✓ **human parechovirus 1 (46 reads)** ✓ Pandoravirus (247 reads) ✓ Actinobacteria (538,844 reads) Bifidobacteriales (463,853 reads) ✓ Firmicutes (101,073 reads)	✓ **Calicivirus (2**,**570 reads)** • G1/10360/2010/NM 950 reads • GI/DH1751/2009/IND 30 reads • GI.3/13440/2007/RJ/BRA 80 reads • GI.3/C9/GF/1978 10 read • GI.4/1643/2008/US 70 reads • GI.4/15waterBS/T11/ITA 10 read • GII.4 Bejing 40 reads ✓ α-retrovirus (150 read) ✓ Parvovirus NIH-CQV (10 read) Bacteria
PathoScope	1,226,383 aligned reads; 2,210 hits ✓ α-retrovirus (113 reads) ✓ HHV8 (7 reads) ✓ Pepper mild mottle virus (2 reads) ✓ Actinobacteria, Bifidobacteriaceae (166,773 reads) ✓ Bacteroidetes (784,119 reads) ✓ Firmicutes, Clostridiales (138,760 reads)	1,116,813 aligned reads, 2,205 hits ✓**Sapovirus_Hu/Dresden/pJG-Sap01/DE (831 reads)** ✓**human parechovirus (19 reads)** ✓α-retrovirus (16 reads) ✓human herpesvirus 6A (2 reads) >900,000 reads Bifidobacterium	16,713,832 aligned reads; 2,558 hits ✓**Norovirus G1 (20**,**880 reads)** ✓human papillomavirus (8 reads) ✓polyomavirus (4 read) ✓Hepatitis C Virus (35 reads) ✓Human Herpesvirus (89 reads) ✓α-retrovirus (2,423 reads) >5,000,000 reads Bacteroides
metaMix	88 hits; 1,771 human reads Bacteria; 658,685 reads Bacteroides Bifidobacterium	45 hits; 0 human reads ✓ **Human parechovirus (1contig; 398 reads)** Bifidobacterium	138 hits; 0 human reads ✓ **Norovirus GI (1 contig; 1**,**169**,**366 reads)** ✓ **Norovirus Hu/GII**.**P16/GII**.**13/New/Taipei/13-BA-1/2013/TW (1contig; 3**,**912 reads)** Circoviridae (1 contig; 5,433 reads)

^*^Files >5GB are not supported by taxonomer BETA version; 10,000,000 reads were randomly sampled to meet 5 GB maximum size for upload. # Genbank entry KX396056 is identical to NC_031324 describing a human norovirus in diarrheic chimps; next closest assignment NC_039897.1, human Norovirus GI, 92% sequence identity.

**Table 4:** Comparison of CSF sample analysis results obtained by DAMIAN, Taxonomer BETA, PathoScope and metaMix.

	7653
DAMIAN	1,618,480 reads 86,86% human sequences
	✓ **Enterovirus B (660 reads**, **1**.**28%)** • **Echovirus 30 (2 contigs; 660 reads) 98% id**.
Taxonomer BETA	1,600,000 reads sampled; 5% classified
	✓ **Enterovirus B (123 reads)** **• Echovirus 30 (49 reads)** • Coxsackievirus B2 (10 reads) ✓ α-retrovirus (742 reads) ✓ Caudovirales (745 reads) ✓ Herpesvirales (57 reads) • HHV6A (21 reads)
PathoScope	6,021 aligned reads; 2,234 hits ✓ **Enterovirus (39 reads)** • Enterovirus 107 (30 reads) • Enterovirus 100 (4 reads) • Enterovirus B (5 reads) ✓ Encephalomyocarditis Virus (85 reads) ✓ Adenovirus (1 read) • Adenovirus F (1 read) ✓ Hepatitis C (1 read) • Genotype 1 (1 read) ✓ α-retrovirus (398 reads)
metaMix	its; 182,276 human reads ✓ **Echovirus 30 (1 contig; 794 reads)**

**Table 5:** Comparison of analysis results for stool samples obtained by DAMIAN, Taxonomer, PathoScope and metaMix.

	SRR533978	SRR1553464	SRR1564804
DAMIAN	2,538,346 reads 7.7% human sequences	1,752,608 reads 1.34% human sequences	627,013 reads 0.83% human sequences
	✓ **Bas Congo Virus (7 contigs**, **8**,**366 reads**, **0**.**6%) 99**.**94% id**. ✓ Paraburkholderia tropica (335,121 reads, 23.9%) ✓ Paraburkholderia fungorium (186,569 reads, 13.3%)	✓ **Zaire Ebolavirus (1 contig**, **1**,**740**,**198 reads**, **98**.**1%) 98**.**45% id**. ✓ Ralstonia (8,482 reads, 489 contigs)	✓ **Equine infectious anemia virus (1 contig 89 reads**, **0**.**02%)** ✓ **GB virus C (2 contigs**, **48 reads**, **0**.**01%)** ✓ **Chlamydophila psittacci (59 contigs; 328**,**399 reads**, **71**.**3%)**
Taxonomer BETA	196 K reads sampled, 23% classified threshold 50 reads	179 K reads sampled, 67% classified threshold 50 reads	184 K reads sampled, 50% classified threshold 50 reads
	✓ **Bas Congo Virus (477 reads)** ✓ **Human Rotavirus A (94 reads)** ✓ Neisseria meningitis 6,334 reads ✓ Mycobacteria (2,814 reads) ✓ Microbacterium laevaniformans (13,314 reads) ✓ Candida albicans (199 reads)	✓ **Zaire Ebolavirus (86**,**872 reads)** ✓ Bradyrhizobium (1,595 reads) ✓ Actinobacteria (1,776 reads)	✓ **Chlamydophila psittacci (4**,**999 reads)** ✓ **GB Virus C (121 reads)** ✓ Actinobacteria (7,830 reads) ✓ Bacilli (4,025 reads) ✓ Alphaproteobacteria (10,169 reads)
PathoScope	537 hits ✓ Burkholderia gladioli BSR 3 (88,777 reads) ✓ Staphylococcus epidermidis (15,834 reads) ✓ Acidovorax sp. JS42 (15,306 reads) ✓ Hepatitis C Virus (58 reads)	843 hits ✓**Ebolavirus Zaire 1976 strain (1**,**309786 reads)** ✓Ralstonia pickettii (12,823 reads) ✓HHV-4 (4 reads) ✓Human Adenovirus C (1 read)	802 hits ✓ **Chlamydophila psittacci (392**,**837 reads)** ✓ Ralstonia pickettii (30,029 reads) ✓Staphylococcus aureus (10,436 reads) ✓**Equine infectious anemia virus (115 reads)** ✓**GB virus C (73 reads)**
metaMix	109 hits; 11,130 human reads ✓ **Bas-Congo Tibrovirus (1 contig; 8**,**849 reads** ✓ Paraburkholderia 11 contigs; 89,485 reads ✓ Burkholderia 24 contigs; 79,270 reads	141 hits; 406 human reads ✓ **Zaire Ebolavirus (1contig; 2**,**230**,**594 reads)** ✓ Ralstonia (9 contigs; 6,090 reads) ✓ Bradyrhizobium (11 contigs; 2,334 reads)	31 hits; 123 human reads ✓ **Chlamydia psittaci (1 contig; 587**,**653 reads)** ✓ **Equine infectious anemia virus (1 contig; 146 reads)** ✓ **GB virus C (1 contig; 45 reads)**

#### Respiratory (BAL) samples

**Figure 2:**
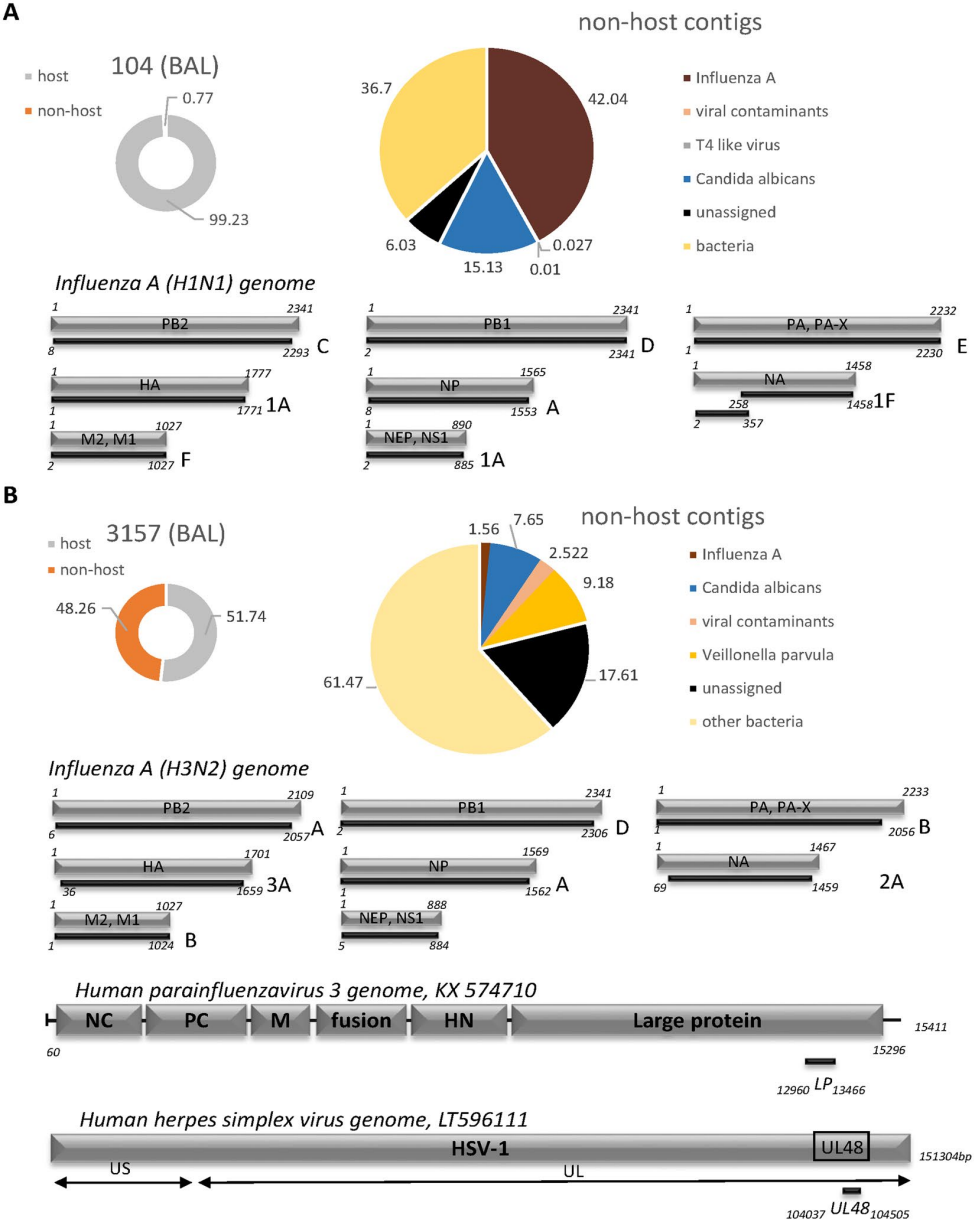
Application of DAMIAN to RNA-Seq libraries from diagnostic BAL samples from patients with viral respiratory infections. Donut shaped charts represent the distribution of host (grey) versus non-host (orange) reads. The pie chart illustrated the taxonomic classification of non-host reads; represented are the relative abundance of contigs assigned to these species. Reads not aligning to sequences in the NCBI database are indicated in black, bacterial sequences are represented in yellow, viral contaminants are shown in pink. The pathogen most likely contributing to the clinical symptoms is indicated in read. In each sample, the contigs of the putative pathogen identified in the sample are aligned to the closest relative: (**A**) Influenza A, H1N1 (full-length segments); (**B**) Influenza A, H3N2 (full-length segments); PIV3 and HSV-1.

**Figure 3:**
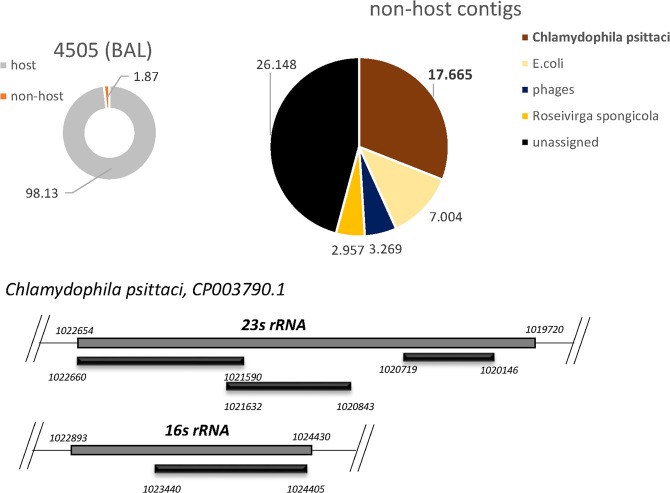
Application of DAMIAN to RNA-Seq libraries from diagnostic BAL samples from patients with bacterial respiratory infections. Similar to Fig. 2, the pie charts represent the distribution of host and non-host reads (left) and the taxonomic classification of non-host reads (right). The contigs of the putative pathogen identified are aligned to the closest relative, *Chlamydophila psittaci*.

DAMIAN readily detected Influenza A in the two routine diagnostic samples investigated in this study (BALs 104 and 3157). The presence of Influenza A was first called after 73 and 40 minutes in samples 104 and 3157, respectively, and inspection of the analysis report identified H1N1 and H3N2 strains as the most likely source of infection (Table 2, Supplementary Datasets S2 and S3). As expected for BAL material, all samples exhibited high abundance of human sequences, with significant variation between the individual samples ranging from approximately 52 to 99% of sequence reads (Figs 2, 3 and Table 2). Routine diagnostic PCRs for a standard panel of respiratory viruses was performed in parallel and yielded positive Ct values of 26 and 30 for Influenza A in BALs 104 and 3157, respectively. All other respiratory viruses included in the PCR panel (hPIV 1–3, hRV, Enteroviruses, Adenovirus, hRSV) were negative (Suppl. Table S1). The significantly lower Ct value observed for Influenza A in BAL 104 is in agreement with the fact that the relative fraction of Influenza A reads was much higher in this sample compared to BAL 3157 (approximately 42 and 1.6%, respectively). The assembled contigs allowed recovery and strain assignments for all influenza genomic segments (Fig. 2A,B, Table 2), thus permitting immediate identification of putative reassortment events between the individual segments. We performed lineage assignment with the FluGenome tool^18^, which reported genotype H1N1 (C (PB2), D (PB1), E (PA), 1A (HA), A (NP), 1F NA), F (MP), 1A (NS)) for sample 104 and H3N2 (A, D, B, 3A, A, 2A, B, 1A) for sample 3157. In addition to Influenza A virus, DAMIAN detected a putative coinfection with *Candida albicans* (15.13% and 7.65% of all non-host reads, respectively; see Fig. 2 and Table 2) in both BAL samples. BAL 3157 also displayed one shorter contig (505nt) unambiguously assigned to the human parainfluenza virus 3 genome (sequence identity 98.75%), and a shorter contig (458nt) with 99.36% identity to human herpesvirus 1 (HSV-1; Fig. 2B, Table 2). The co-infections with both *Candida albicans* and parainfluenzavirus 3 were confirmed by conventional diagnostic methods (fungal culture and PCR). We also included a third BAL sample (BAL 4505) which was one out of three samples of a suspected infectious disease outbreak published earlier^2,6^ in our analysis. In accord with our previous results, DAMIAN correctly identified *Chlamydophila psittaci* and assigned 4.13% of all non-host reads to rRNA moieties originating from the intracellular bacterium (Fig. 3, Table 2).

The comparative analysis results obtained with Taxonomer, PathoScope and metaMix for the three BAL sample datasets are shown in Table 2. While all tools identified *Chlamydophila psittac*i in sample BAL 4505, they differed substantially in the number of assigned reads (237 reads for DAMIAN, 75 reads for Taxonomer, 23.83 reads for PathoScope and 252 for metaMix). The same was true for Influenza A in sample 104 (1,227, 12,234 and 46,698 reads, respectively). Only DAMIAN was able to assign the correct genotype and strain for each individual segment. PathoScope and Taxonomer were both unable to differentiate between H1N1 and H3N2 in samples 104 or 3157, respectively. MetaMix correctly assigned H1N1 to one contig. Furthermore, the observed co-infections of *Candida albicans* and parainfluenzavirus 3 were only identified by DAMIAN or Taxonomer for sample 3157 (Table 2), whereas co-infections in sample 104 were detected by DAMIAN and metaMix.

#### Stool samples

**Figure 4:**
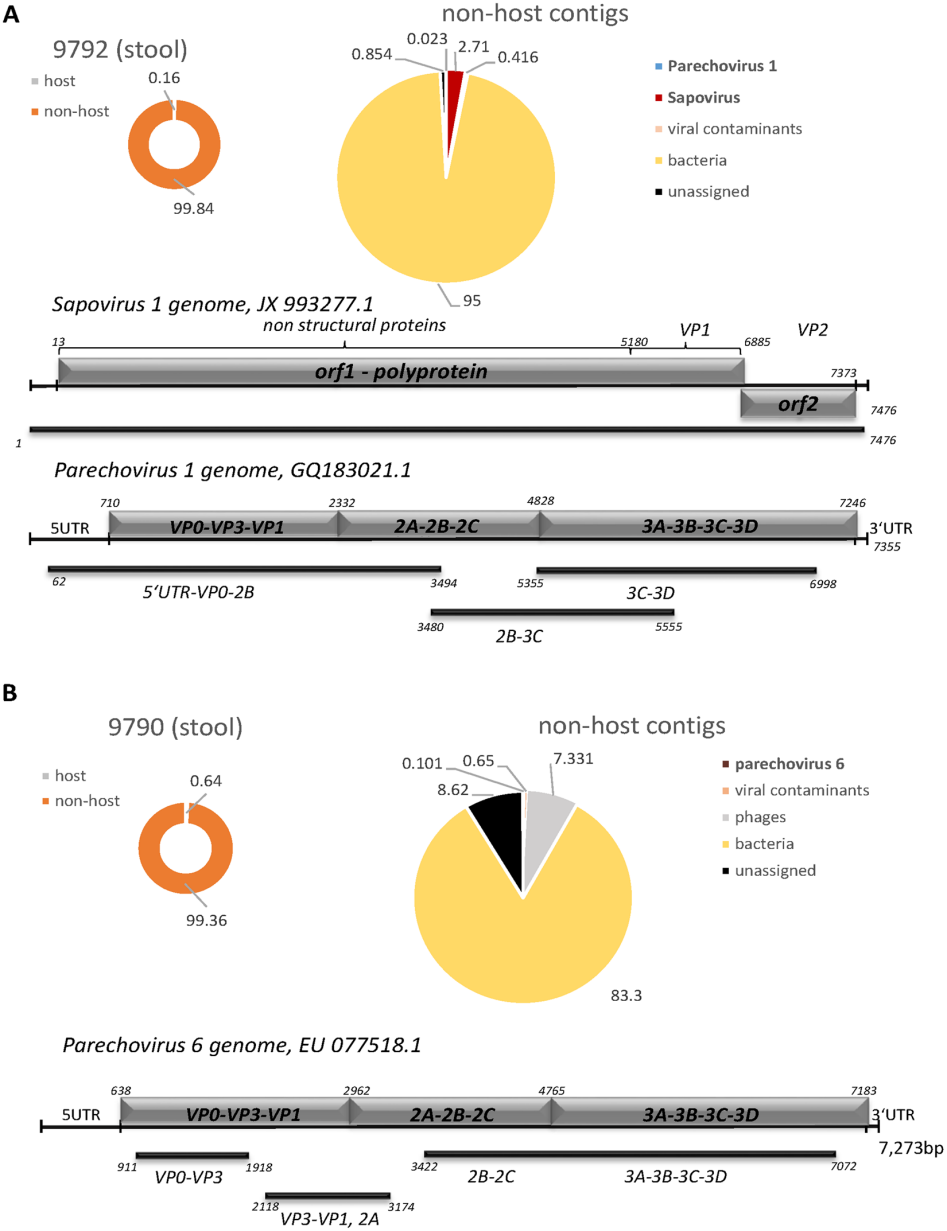
Application of DAMIAN to RNA-Seq libraries from diagnostic stool samples from patients with acute gastroenteric disease. Sequences are depicted as described in Fig. 2. The putative pathogens identified are (**A**) Sapovirus 1, Parechovirus 1 and (**B**) Parechovirus 6.

**Figure 5:**
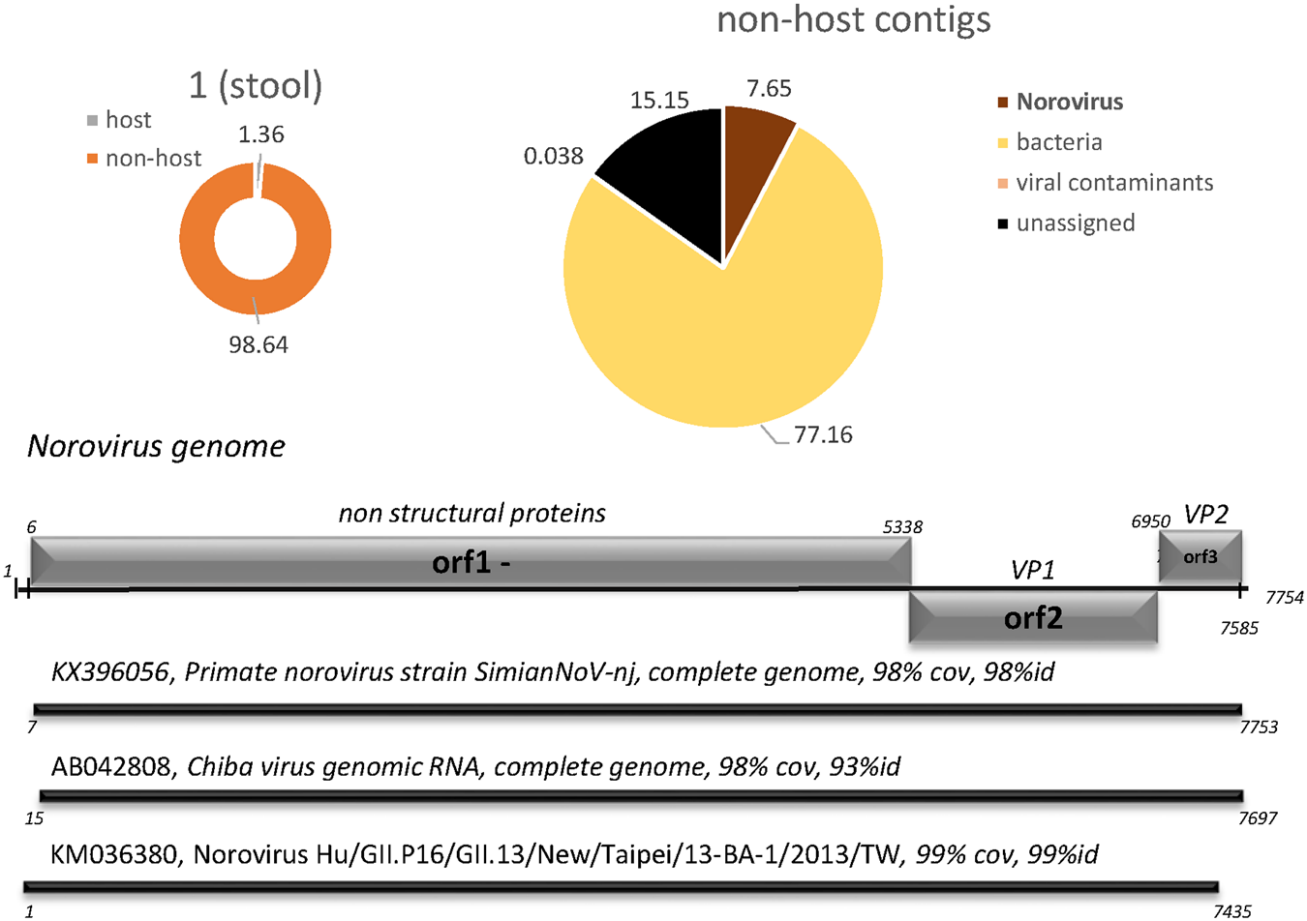
Identification of full-length genomes of three different Norovirus strains from a stool sample from a patient with acute gastroenteric disease. Primate Norovirus, Genbank entry KX396056 is identical to NC_031324 describing a human norovirus in diarrheic chimps; next closest assignment NC_039897.1, human Norovirus GI, 92% sequence identity.

We included three stool samples collected during a large outbreak of acute gastroenteritis (AGE) occurring in fall of 2012 in Germany^19,20^, in our comparative analysis (Figs 4, 5 and Table 3). RNA from two samples (9790 and 9792) was sequenced with approximately 1.5 million reads per sample on a MiSeq instrument, while RNA material from the third (sample 1) was sequenced at a depth of 23.3 million on a HiSeq instrument. As expected for most stool samples^21^, only few host sequences were present (generally between 0.2 and 1.4%). Contigs aligning to caliciviral sequences were assembled in two of the three libraries: Sample 1 contained Norovirus (hNoV) sequences, whereas Sapovirus sequences were detected in sample 9792. In both cases, contigs representing complete or near-complete caliciviral genome sequences were recovered. In sample 1, inspection of the contigs furthermore readily revealed co-infection with three Norovirus strains. Sequences were assigned to two different genotype I strains (98.35% and 92.91% sequence identity to primate norovirus strain Simian NoV-nj (gb|KX396056) and the next closest relative, Chiba virus (gb|AB042808), respectively), and a third contig representing recombinant norovirus of genotype GII.16/GII.13 with 98.79% sequence identity to the Taipei/13-BA-1 isolate (gb|KM036380) (Fig. 5).

Interestingly, samples 9790 and 9792 also contained reads from picornaviruses with significant nucleotide homologies to human parechovirus type 6 (hPeV6) or human parechovirus type 1 (hPeV1) (Table 3). Sample 9792 yielded three contigs of 1,648; 2,130 and 3,463 nt covering approximately 95% of the most closely related hPEV1 strain (97.44%, 90.59% and 97.22% sequence identity to isolate 550163, accession GQ183021.1, respectively). In sample 9790, contigs of 1,010; 1,063 and 3,650 nt aligned to approx. 80% of human parechovirus type 6 (isolate 2005-823, accession EU077518.1) with 96.43%, 95.83% and 96.63% sequence identity.

Similar to the respiratory samples, the stool sample datasets were also analyzed by Taxonomer, PathoScope and metaMix. Results are summarized in Table 3. DAMIAN, Taxonomer and PathoScope tools identified Sapovirus GI together with human parechovirus in sample 9792, but only DAMIAN and Taxonomer specified the human parechovirus as a type 1 strain. However, metaMix did not identify Sapovirus under the conditions used. The tools identified different Sapovirus strains (see Table 3) with DAMIAN identifying Sapovirus Hu/G1/BE-HPI01/DE/2012, the sequence which was originally identified with DAMIAN from this sample and submitted to Genbank (accession number JX993277.1). Taxonomer reported Sapovirus Hu/GI.2/BR-DF-01/BRA/2009 and PathoScope listed Sapovirus Hu/Dresden/pJG-Sap01/DE (GenBank accession number NC_006269.1) instead, with the latter showing 73% sequence identity and 84% coverage to the original Hu/G1/BE-HPI01/DE/2012 sequence present in sample 9792.

DAMIAN, taxonomer both identified human parechovirus sequences in sample 9790. However, the three contigs assembled by DAMIAN unequivocally aligned to human parechovirus type 6, whereas Taxonomer assigned 46 sequence reads to human parechovirus type 1. PathoScope and metaMix did not detect any parechovirus sequences in sample 9790 (Fig. 4, Table 3) at all.

The fact that DAMIAN assembled full-length contigs for 3 different norovirus genotypes in sample 1 suggests that this patient acquired an infection in the course of the 2012 norovirus outbreak, the largest recorded food-borne outbreak in Germany with more than 4,000 cases registered by the public health agencies^19,20^. Most of the samples analyzed during this outbreak showed co-infection with multiple Norovirus genotypes, indicative of massive fecal contamination of food sources representing the origin of the outbreak^19,20^. In accordance with the public health data, DAMIAN recovered two discrete full-length Noroviruses of genotype I as well as a recombinant GII.16/GII.13 genome from the sample. Together, over one million reads were mapped to the three genomes. MetaMix successfully classified two contigs as Calicivirus sequences of genotypes GI and recombinant GII.16/GII.13, with the GI sequence being much more abundant compared to the recombinant genotype II. In contrast, Taxonomer assigned 2,570 reads to seven different Norovirus strains of genotypes I and II, whereas PathoScope classified 20,880 reads as originating exclusively from norovirus genotype I (Table 3).

#### CSF samples

**Figure 6:**
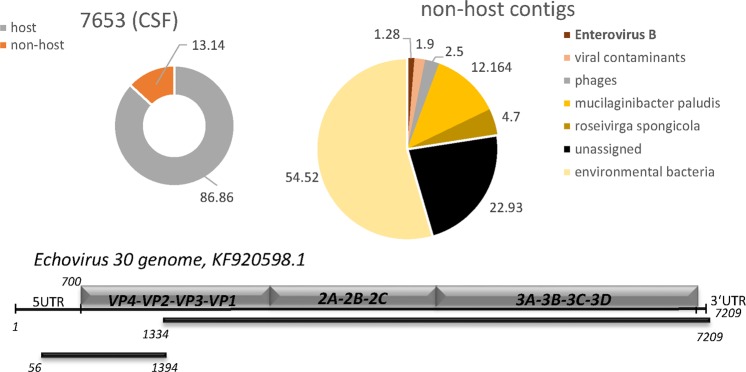
Application of DAMIAN to RNA-Seq libraries from diagnostic stool samples from patients with encephalitis. Sequences are depicted as described in Fig. 2. Two contigs representing significant sequence homology to Echovirus 30 were identified.

We included one routine diagnostic CSF sample in the comparison. The sample was submitted by the clinic with the request to detect viruses known to induce encephalitis in immune competent patients. Parallel to quantitative PCR for HSV, Enteroviruses, Mumps, Measles and Rubella, the sample was analyzed by DAMIAN, Taxonomer, PathoScope and metaMix. DAMIAN and metaMix both reported Echovirus 30, a call that is concordant with results obtained by diagnostic PCR (Supplementary Table S1) and subsequent Sanger sequencing of the 250 bp fragment. Two contigs covering nearly the complete genome, were recovered (Fig. 6). Taxonomer identified 123 reads as Enterovirus B, with 10 reads assigned to Coxsackievirus B2 and 49 reads to Enterovirus 30. PathoScope identified Enterovirus sequences (39 reads in total), however none of the reads was assigned to Echovirus 30 (Table 4).

#### SRR samples

In addition to the the diagnostic samples collected in this study, we applied DAMIAN to three datasets (SRR533978, SRR1553464 and SRR1564804) which have been which have been used by Flygare and colleagues to evaluate the ability of Taxonomer to detect viruses in public health emergency samples^11^. Similar to our analysis of CSF, stool and BAL samples we compared the DAMIAN results of these datasets to those obtained with Taxonomer, PathoScope and metaMix (Table 5, Supplementary Fig. S1A–C and Suppl. Datasets S9–S11). SRR533978 represent RNA-Seq data from a serum of a patient with hemorrhagic fever caused by Bas Congo Virus (Suppl. Fig. S1A). SRR1553464 is a plasma sample from a patient with Ebola virus infection (Suppl. Fig. S1B), and SRR1564804 represent a plasma sample from a patient with *Chlamydophila psittaci* infection (Suppl. Fig. S1C). All tools identified Bas Congo Virus in sample SRR533978, Ebolavirus Zaire in SRR1553464 and *Chlamydophila psittaci* as well as GB-Virus C in SRR1564804. In the case of the viral infections, DAMIAN recovered whole viral genomes for Bas Congo Virus (7 contigs, 467 bp–4,977 bp) and Ebolavirus (1 contig, 18,839 bp). GB-Virus C in sample SRR1564804 was only represented by two small contigs of ~600 bp, indicating it may have been present in relatively low copy numbers. Detection of *Chlamydophila psittaci* in the sample was based on contigs aligning to 16S and 23S rRNA. Differences between the individual tools were observed with regard to the number of reads assigned to the individual taxons. In addition, only DAMIAN, PathoScope and metaMix identified equine infectious anemia virus in SRR1564804.

### Cohort based analysis

#### Identification of pathogen signatures shared among outbreak samples

**Figure 7:**
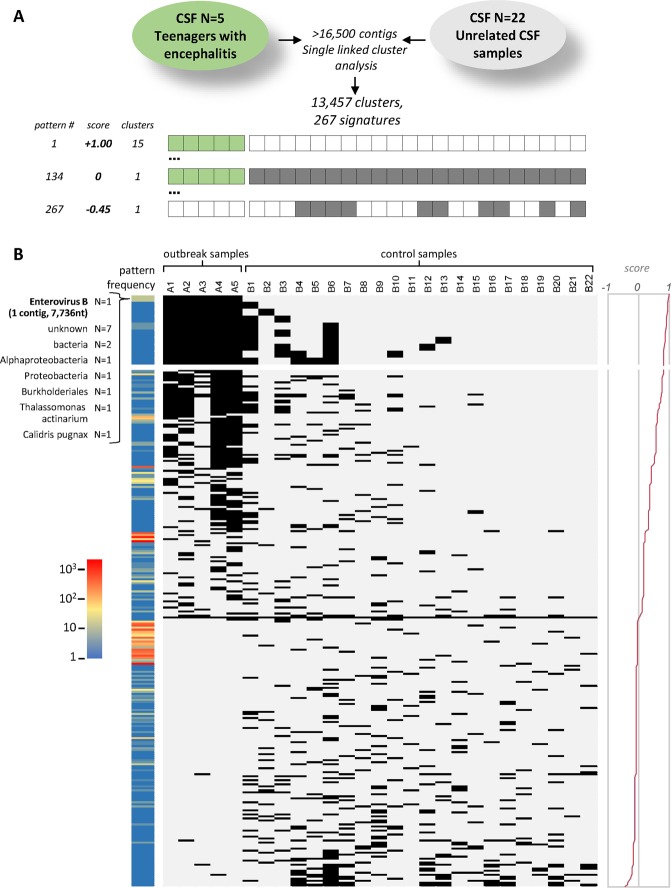
Cluster analysis of CSF samples from encephalitis and control cases. (**A**) Schematic depiction of the cohort analysis performed on five samples derived from an enterovirus outbreak and 22 unrelated control samples. Single linked cluster analysis produced 13,457 clusters from ~16,500 individual samples. Depending on the distribution of samples that do or do not contribute contigs to a given cluster, these can be assigned to one of a total 267 observed ‘signature’ patterns. The lower panel schematically depicts the highest (score = + 1), lowest (−0.45) and neutral (0) scoring signature patterns, with filled (dark green or grey for encephalitis or cohort samples, respectively) or empty squares symbolizing samples that do or do not contribute contigs, respectively. The total numbers of clusters assigned to each of the three signatures is shown to the left. (**B**) Distribution map and frequencies of observed signature patterns. Each row depicts one of the 267 observed signature patterns as described above under (**A**). Signatures (black and light gray rectangles for positive and negative samples, respectively) are ordered by their score (plotted to the right). The ten signatures in which all encephalitis samples contribute contigs are shown enlarged at the top. The colored heat map bar to the left indicates the number of clusters that share a given signature pattern. The taxonomic annotation (lowest common ancestor of individual contig assignments, or ‘unknown’ if contigs do not have significant hits) of the 15 clusters with the highest scoring pattern are indicated at the top.

To demonstrate the ability of the cohort-based analysis tool to identify pathogens that may be responsible of infectious disease outbreaks, we analyzed five CSF samples derived from an enterovirus meningitis outbreak occurring in the Hamburg region during summer 2015 (Supplementary Table S1). CSF samples were negative by diagnostic PCR for HSV, VZV, EBV and *Borrelia burgdorferi* while samples showed Ct values between 31 and 33 for Enterovirus B PCR. As a negative control group in our cluster analysis, we used 22 unrelated routine diagnostic CSF samples that had tested negative in diagnostic taqman-PCR for a panel of viruses commonly involved in encephalitis. Table S3 summarizes the sequencing data of all samples included. Figure 5A depicts a schematic outline of our analysis. In total, more than 16,500 contigs were assembled across the 27 samples. The single linked cluster analysis tool integrated in the DAMIAN pipeline (see Material & Methods for details) produced 13,457 sequence clusters from these contigs. For each individual cluster, the fraction of positive samples in the outbreak and control cohorts was determined, and a cluster score was calculating by summation of the positive outbreak fraction value and negative value of the control fractions. Accordingly, the resulting score can take a maximum value of +1 if all samples in the outbreak cohort are positive while all controls are negative, or minimally reach a value of −1 if all control but no outbreak samples are positive. Overall, we observed 267 discrete patterns of positive and negative samples among the 13,457 sequence clusters, with scores that ranged from +1.00 to −0.45. A map of all signature patterns (sorted by descending score) along with their observed frequencies is shown in Fig. 7B. The full distribution of clusters and assignment of sequences within the cluster can be found in Supplementary Dataset S1.

Overall, a total of 30 sequence clusters were shared among all five outbreak samples; of these, 15 were not present in any of the control samples and consequently were awarded the highest score of +1.00 (see annotated top-scoring pattern in Fig. 7B and Supplementary Dataset S1). Only one of these fifteen clusters was assigned to a pathogenic species, namely Enterovirus B. Interestingly this cluster contained 14 contig sequences, with the longest contig encompassing 7,337 nt (and thus extending over the entire length of the Enterovirus B genome). The contig contained one single ORF with proteins clearly identified as Enterovirus protein domains (see Supplementary Dataset 1). The other eleven clusters were either of environmental or commensal bacterial origin (n = 6), unknown origin (no match in NCBI database, n = 4) or unclear origin (Calidris pugnax, n = 1). Thus, while DAMIAN readily classified the assembled Enterovirus B contigs taxonomically due to their nucleotide homology to existing NCBI database entries, even if the taxonomic classification had failed the approach presented here would have reduced the number of candidates that may be responsible for the outbreak to just a handful.

#### Reoccurring viral contaminants

In addition to its value for identifying putatively novel pathogens, the cohort based analysis tool is also useful to identify and flag common contaminants that are frequently present in NGS data. Such contaminants, for example, may reflect environmental bacteria that are introduced by excessive handling of the diagnostic specimen. In addition, contaminants may be introduced via laboratory materials and reagents, for example, retroviral sequences that originate from reverse transcriptase enzyme preparations in library kits, or parvoviral sequences that likely stem from silica gel columns used for nucleic acid extraction^14,22,23^. By virtue of the fact that they register in all (or nearly all sequences), such sequences can be easily identified by DAMIAN, and subsequently can be excluded from downstream analyses. By default, DAMIAN filters for a number of viral sequences (mostly representing unclassified circular DNA viruses; see complete list in Supplementary Table S2) that we have frequently detected in our metagenomic DNA or RNA shotgun sequencing experiments. These sequences are identified by DAMIAN and flagged as putative contaminants in the DAMIAN output files (for examples, see entries in light grey color code in Supplementary Datasets S2–S11). To our knowledge, no other tools aimed at diagnostic NGS applications recognize such contaminants. For example, both PathoScope and Taxonomer report alpharetroviral sequences in BAL sample 104, whereas DAMIAN clearly flags the corresponding contigs as putative contaminants (Supplementary Dataset S2).

## Discussion

DAMIAN is a publicly available, comprehensive software tool for the fast and reliable detection of pathogens specifically in diagnostic samples. To our knowledge, it is the first software to include a tool for cohort based analyses, a feature which can be highly valuable in infectious disease outbreak scenarios where multiple samples have to be compared for presence of shared pathogen sequences. DAMIAN is easy to use and easy to install. Its output provides an interpretation of its findings (including flagging of commensals and technical artifacts) and allows for fast decision making in clinical context. Assembled sequences, which often represent complete or near-complete viral genomes, are a part of the output. DAMIAN automatically documents its analyses. Software and database versions, parameters and similar information is stored and allows to quickly describe or reproduce an analysis.

Using primary/authentic diagnostic samples that have been well characterized by conventional diagnostic (culture and PCR) methods (Figs 2–6), as well as publicly available benchmarking data sets originally used to validate the Taxonomer pipeline (Table 5; Supplementary Fig. S1 and Supplementary Datasets S9–S11), we have verified that DAMIAN accurately identifies viral and bacterial pathogens. Furthermore, DAMIAN allows reliable classification of viral sequences at the species level and, in most cases, even at the strain level. Compared to DAMIAN, the other tools tested here provided strain level assignments which were substantially more error prone or incomplete. This is especially true for those tools, which are based on classification of single reads (PathoScope, Taxonomer). For example, only DAMIAN was able to assign Sapovirus, Chiba Virus and Norovirus strains in human stool samples. DAMIAN is furthermore superior in detecting and differentiating between individual strains of multiple viral species present in a single sample, as demonstrated by the analysis of a stool a sample (sample 1) originating from a large AGE outbreak in Germany that had been caused by sewage-contaminated food sources. Indeed, DAMIAN was not only able to identify the individual strains, but also assemble complete (or near-complete) genomes of the GI and GII Norovirus genotype viruses, a feature which is highly valuable when investigating infectious disease outbreak situations such as the 2012 AGE outbreak^19,20^.

Of note, while the data presented in Tables 2–5 demonstrate complete or near complete recovery of RNA virus genomes (or genome segments) with a size of 20 kb or less, DAMIAN is also able to assemble considerably larger viral genomes. For example, we recently used a previous version of the pipeline to help recover the full sequence of a novel seal parapoxvirus from DNA-seq reads derived from a skin lesion^24^. In Supplementary Fig. S1D and Dataset S12 we furthermore demonstrate that RNA-seq reads can be used to recover near-complete DNA-virus genomes. In this case, unbiased RNA sequencing of a human stool sample from an immunosuppressed patient allowed recovery of 12 contigs (1,929 bp to 10,132 bp) which covered the full genome of human adenovirus type 31. Of course, successful assembly of complete DNA viruses from RNA-seq reads will require abundant transcription across the majority of the viral genome. Hence, RNA-seq of samples in which viral transcription is restricted (e.g., latently infected cells) are very unlikely to yield complete viral sequences.

The possibility to perform cohort-based analysis of multiple samples represents a unique advantage of the DAMIAN pipeline. Independent of taxonomic classification, this tool allows the identification of sequence signatures that are uniquely (or preferentially) associated with a given sample (e.g., disease-associated) cohort when compared to a collection of control samples. While information from external database can be integrated, the main advantage of this approach is that such information is not at all required to detect pathogenic agents.

We have previously used a similar approach to help resolve a suspected outbreak involving three patients suffering from severe pneumonia. As initial routine diagnostics failed to detect an infectious agent, it was speculated that the cases may represent an outbreak of a novel pathogen. Upon NGS-based analysis of BAL material, however, our pipeline readily called the presence of *Chlamydophila psittaci* in one of the samples, an infection which was subsequently confirmed by routine diagnostic procedures as the cause of the observed clinical symptoms. Importantly, neither on the level of taxonomic assignments nor after performing pairwise BLAST alignments did we find any evidence of a potential shared pathogen sequence signature among the three samples, strongly arguing against the hypothesis that the cases represented an outbreak of a novel pathogen^6^.

While the above example highlights the usefulness of combining taxonomic assignment with cross-sample sequence alignments to rule out an infectious disease outbreak, we here also demonstrate the ability of the DAMIAN cohort analysis tool to identify a causative pathogen in authentic outbreak samples. Remarkably, the assembled Enterovirus B genomes represented one of only a handful of clusters that were shared among all five outbreak samples, but were not present in the control cohort. Notably, while Enterovirus B was also identified taxonomically, the clustering result *per se* is completely independent of taxonomic classification. Even if Enterovirus sequences were not present in the database (or if no reference databases were available at all), it would be fairly straightforward to hunt for the causative agent among the top-scoring fifteen candidates that were ranked solely due to their pairwise sequence homology across the sample cohorts.

Naturally, depending on the given type of disease or diagnostic specimen it will not be always feasible to presume that a causative pathogen must be present in 100% of the outbreak samples at the time of diagnosis, while being completely absent from the controls. Even in such scenarios, however, ranking of the contigs according to the scores awarded by the cohort analysis tool will allow identification of those sequences which are preferentially associated with a given disease cohort. Hence, especially in cases where the presence of a potentially novel pathogen is suspected, we expect that researchers as well as clinicians will find DAMIAN a valuable tool to help eliminate contigs originating from common microorganisms or contaminants, and thus aid in focusing on those sequences that represent the most promising candidates for a causative pathogen.

## Materials and Methods

### Quality control

Trimmomatic^25^ was integrated for the optional removal of low quality bases and sequencing adapter sequences. DAMIAN executes the program with predefined parameters, which can be modified. Information on read properties prior and after this step is collected by DAMIAN and stored in its database.

### Digital subtraction and abundance estimations

Digital subtraction and abundance estimation of unwanted sequence reads is optional and DAMIAN can be used with any number of different host reference genomes or no host genome at all. Bowtie2^26,27^ was integrated for read alignment tasks. Host abundance estimation is performed on a subset of sequence reads (default 1 M reads) using Bowtie2’s ‘sensitive-local’ parameter preset. Reads aligning without insertions and deletions and with a minimal mapping quality of 10 are used to estimate the size of sequenced fragments and its standard deviation. Digital subtraction is performed on all reads. Here the ‘fast’ preset is applied, which enforces end-to-end alignments. Bowtie2, like all other tools, was integrated and the user is not required to be familiar with its functionality. Sequence indices, for example, are built automatically.

### Assembly and assessment of basic contig features

Sequence reads are assembled using IDBA-ud^28^. Following its author’s instructions, the source code of the program was slightly modified to support reads up to a length of 250 bp. DAMIAN processes the assembled contigs individually. It extracts open reading frames by translating the contig sequences in the six possible reading frames and subsequently identifying putative amino acid sequences of a given minimal length (75 bp per default) which are not interrupted by stop codons. Sequence complexity is assessed using dustmasker from the NCBI Blast + suite. Contig abundance is calculated based on the alignment of sequence reads to the contigs. This task is performed with Bowtie2. Coverage tracks for every contig are stored in the database.

### Functional annotation and contig ranking

Derived amino acid sequences are screened for known protein domains using HMMER^29^ and the PFAM^30^ database. DAMIAN classifies PFAM domains according to their taxonomic occurrences. Asides from being an additional level of evidence for the detection and classification of pathogens, the domain annotation is also used to determine the order in which contigs are processed in the subsequent analysis steps. Contigs are ranked according to the number of bases located in annotated domains and according to whether or not these domains are known to be exclusively present in viruses. Reordering the contigs does not affect the results, but since DAMIAN reports important findings immediately and since preliminary results in Excel-format can be generated before an analysis is finished, the ranking leads to putative pathogens being reported earlier.

### Taxonomic assignment

Ranked contigs are processed with BLAST^31^ to identify similar sequences in NCBI’s nt and nr database. The default strategy is to perform only MEGABLAST searches. Optionally BLASTN and BLASTP can each be used on every contig or only on contigs, which did not yield a match with the preceding less sensitive search. For every contig, all matches with a bitscore at least as high as 90 percent of the highest observed bitscore for a given contig, are stored in the database. Additionally, NCBI’s taxnames and taxnodes are used to traverse taxonomic lineages and determine the lowest common ancestor (LCA) for the matches with the single highest bitscore and, separately, for those matches in the stratum defined above. If the LCA is a viral taxon and neither a phage nor a deliberately excluded taxonomic entity, then it is being reported immediately. Once all contigs have been processed with BLAST, the initial analysis is complete. At this point, final reports can be generated for individual samples or a cohort of previously processed samples can be analyzed jointly.

### Refined taxonomic assignment and reporting

For the report of individual samples, contigs are taxonomically assigned a second time using a strategy which incorporates information from all contigs. First, all species observed in any BLAST match of any contig are ranked according to the bitscore of the matches and abundance of the contigs yielding these matches. Then every contig is assigned to the highest scoring species it yielded a match for. This strategy follows the assumption that if a contig C1 can be unambiguously assigned to a species S1 and another contig C2 could equally well be assigned to species S1 or S2 then the most parsimonious explanation for this observation is, that both C1 and C2 originate from species S1. Only in rare cases, where this algorithm does not yield an unambiguous match on species level, an LCA is computed from all optimal matches. The output contains, the taxonomic assignment based on the procedure described above, the preceding LCA assignment and the underlying initial BLAST assignments.

### Cohort analysis

The user assigns any number of samples to a group of known positives, known negatives or of unclassified samples. For example all samples which belong to one outbreak can be assigned to the group of positives while samples which are known to be unrelated the outbreak would be assigned to the group of negatives. Finally, samples for which it is unsure whether they are part of the outbreak could be put in the group of unclassified samples. Contigs not meeting user-defined criteria can be excluded from the analysis. These criteria include the information content, the length of the contig, the number of detected protein domains, the number of ORFs and the taxonomic assignment. Remaining contigs are used in an all-versus-all BLAST. From the pairwise results, a (bit-)score matrix is calculated. Single-linkage clustering is performed until the score of the two most similar pair of clusters is lower than a defined threshold. After clustering, a score is calculated for each cluster. The score is based on the number of contigs belonging to each of the three groups and group specific predefined weight, such that clusters with a higher score contain more sequences from the group of positive sample and less sequences from the group of the control group.

Results are reported in spreadsheet format and additionally FASTA files are generated from contig sequences for each cluster (see exemplary results in Supplementary Dataset S1).

### Implementation and availability

The software was written in Ruby and meant to be deployed in Linux environments. A PostgreSQL database is used to store analysis results and associated metadata. Results presented in this publication were achieved using DAMIAN’s standard settings. DAMIAN’s source code is available at https://sourceforge.net/projects/damian-pd.

### Taxonomer

Taxonomer analyses were performed using the web based metagenomics analysis tool provided on http://taxonomer.iobio.io/.

### PathoScope

PathoScope analyses were performed using version 2.06 with default parameters. The optional PathoDB, and PathoReport modules were included and the optional PathoQC module was omitted.

### metaMix

Sequence reads were assembled with IDBA-UD. Contigs longer than 399 bp were aligned to the NCBI nt database with MEGABLAST. Read length and taxon identifiers were incorporated in the BLAST output as described in the metaMix user guide. Finally, metaMix v0.3 was run with standard settings^17^.

### Diagnostic sample

Samples were collected during routine diagnostic analysis performed at the UKE. Respiratory BAL samples derived from patients with respiratory illness and suspected influenza infection. All samples were screened by standard diagnostic quantitative RT-PCR for known respiratory pathogens.

Stool samples, collected during the gastroenteritis outbreak in Germany, were received from the Robert Koch Institute (RKI) and from the DRK hospital (Berlin).

CSF samples were received from the Department of hematopoietic stem cell transplantation. The CSF samples were collected due to neurological complications in these patients. All samples were screened for pathogens involved in CNS infections applying conventional diagnostics. Only samples tested negative were included here. The five CSF samples from teenager with encephalitis were received from the UKE. The study was approved in compliance with relevant laws and institutional guidelines by the local ethics committee, Freie Hansestadt Hamburg, WF-012/15; WF-026/13; WF025/12. The study was conducted retrospectively on anonymously stored clinical samples. Information which would allow the identification of the patient (human sequences, name, address, birth date, hospitalization number) was removed. Under these conditions the ethics committee approved the study on diagnostic samples without an informed consent.

Datasets SRR533978, SRR1553464 and SRR1564804 are derived from SRA, sequence read archive: SRX173233, SRX674125, SRX691917.

### Diagnostic PCRs

The PCR primers and specific probes for influenza virus quantitative PCR used have been described previously^6,32–38^. The following primers and probes were used: Infl.A_F: GACAAGACCAATCCTGTCACYTCTG, Infl.A_R: AAGCGTCTACGCTGCAGTCC, HEX-TTCACG-CTCACCGTGCCCAGTGAGC-BHQ2 and Infl. B_F: TCGCTGTTT-GCAGACACAAT, Infl. B_R TTCTTTCCCACCGAACCA, Cyan500-AGAAGAT-GGAGAAGGCAAAGCAGAACT-DB. Norovirus PCR was performed individually for NoV GI and GII sequences. The following primer and probe sequences were used for GI PCR: NV192 (s) 5′-GCYATGTTCCGCTGGATGC, NV193 (as) 5′-CGTCCTTAGACGCCATCATCA, TM9-MGB probe 5′-VIC-TGGACAGGAGATCGC-MGB-NFQ. For GII PCR the primer sequences NV107c (s) 5′-AICCIATGTTYAGITGGATG and NV119 (as) 5′-TCGACGCCATCTTCATTCAC were used together with the MGB probe TM3AP 5′-6′FAM-TGGGAGGGCGATCGCAATCTGGC-MGB-NQF. Sapovirus PCR was performed using SaV124F 5′-GAY CAS GCT CTC GCY ACC TAC, SaV1245R 5′-CCCTCCATYTCAAACACTA; SaV124TP FAM-CCR CCT ATR AAC CA-MGB-NQF. PCR reactions were performed using the Quantifast pathogen RT-PCR Kit + IC (Qiagen). 5 µl eluate was amplified (Roche Lightcycler 480 instrument) using the following conditions: 20 min @50 °C, 5 min @95 °C, 45 × 15 sec @95 °C, 30 sec @ 60 °C.

### RNA extraction

Stool samples were homogenized in 1.4 ml DNA/RNA buffer (ZR Viral DNA/RNA Kit, Zymo Research) using MP matrix C tubes (Millipore) applying 2 × 30 s at 6,000 rpm in a Precellys 24 tissue homogenizer. Cleared supernatant was transferred to the column and nucleic acid was extracted following manufacturer’s instructions. Nucleic acid was eluted in 30 µl DNAase/RNAse free water.

BAL samples and CSF samples (200 µl) were automatically extracted using QIASymphony (Qiagen Hilden)^6,31^. Nucleic acid was eluted in 100 µl final volume.

### Library preparation and high-throughput sequencing

RNA Illumina NGS libraries were prepared from each sample. Illumina library from RNA was generated using a modified protocol of the SCRIPT SEQTM v2 RNA Seq Kit (Epicentre Biotechnologies) which was described recently^6,33^. All libraries were multiplexed sequenced on an Illumina HiSeq. 2500 instrument (300 cycles, 2 × 150 bp on a paired-end protocol) or MiSeq (300 cycles) according to the manufacturer’s protocol.

## Supplementary information

supplementary material

Dataset S1

Dataset S2

Dataset S3

Dataset S4

Dataset S5

Dataset S6

Dataset S7

Dataset S8

Dataset S9

Dataset S10

Dataset S11

Dataset S12
