# Supplementary material for: DAMIAN: an open source bioinformatics tool for fast, systematic and cohort based analysis of microorganisms in diagnostic samples
Source: Sci Rep. 2019 Nov 14;9:16841. doi: 10.1038/s41598-019-52881-4 (PMC6856179; doi:10.1038/s41598-019-52881-4)

DAMIAN: an open source bioinformatics tool for fast, systematic and cohort based analysis of microorganisms in diagnostic samples.

Malik Alawi, Lia Burkhardt, Daniela Indenbirken, Kerstin Reumann, Maximilian Christopeit,

Nicolaus Kröger, Marc Lütgehetmann, Martin Aepfelbacher, Nicole Fischer and Adam Grundhoff

Supplementary Material

Supplementary Table S1: Clinically relevant PCR results obtained in conventional diagnostics for all clinical samples.

Supplementary Table S2: Viral contaminants observed in unbiased metagenomics sequencing of RNAs (UMERS).

Supplementary Table S3: number of reads and % host reads in samples included in the cluster analysis.

Supplementary Figure S1:
Application of DAMIAN to training datasets obtained from taxonomer reflecting patients samples (A-C) (15) (A) Dataset SRR533978, serum from a patient with hemorrhagic fever in the Democratic republic of Congo; (B) dataset SRR1553464, serum from a patient with suspected Ebola Virus infection in Sierra Leone; (C) dataset SRR1564804, plasma from a patient with a unrecognized *Chlamydophila psittaci* infection. Donut shaped charts represent the distribution of host (grey) versus non-host (orange) reads. The pie chart illustrated the taxonomic classification of non-host reads; represented are the relative abundance of contigs assigned to these species. Reads not aligning to sequences in the NCBI database are indicated in black, bacterial sequences are represented in yellow, viral contaminants are shown in pink. The pathogen most likely contributing to the clinical symptoms is indicated in read.
In each sample, the contigs of the putative pathogen identified in the sample are aligned to the closest relative: (A) Bas Congo Virus; (B) Ebola Zaire Virus, (C) *Chlamydophila psittaci* and
Supplementary Figure S2:
Application of DAMIAN to a stool sample from a immunosuppressed patient with diarrhea.
The identified virus as a putative cause of diarrhea is aligned to its closest relative Adenovirus type 31.

Supplementary Dataset S1: Full distribution of clusters and assignment of sequences obtained from all CSF samples.
Clusters are ranked according to their score. The score is displayed in the first column and its calculation is based on the number of contained affected ('num_pos'), control ('num_neg') and unassigned ('num_oth') samples and specific, user-definable weight. Results presented here were obtained by applying a weight of 1 to affected samples and a negative weight of 2 to samples of the control group. Since five affected samples were present, 5 is the maximum score reachable under the given conditions. The following two columns show the accumulated length of all contigs in a cluster and the number of ORFs they contain. Clusters are stored as multiple FASTA files and the corresponding file name is given in the column 'filename'. The lowest common ancestor of all species present in a cluster according to the original taxonomic analysis of individual samples is shown in the colums labeled 'lca_name (db)' and 'lca_taxid (db)'. If desired cohort analysis ((for example because reference databases have been updated sincce the original analysis) taxonomic assignments may be re-evaluated during the cohort analysis. The corresponding results of this optional step are displayed in the columns 'lca_name (db)' and 'lca_taxid (db)'. Protein domains detected in the sequences of a cluster a listed in the column 'domains'. Finally, the names of individual samples contributing to a given cluster in each group ('positive samples', 'negative samples' and 'other samples') are shown in the last three columns.

Supplementary Datasets S2-S11: Output datasets (Excel workbook format) of the clinical samples generated by DAMIAN:
Suppl. Dataset S2: BAL 104
Suppl. Dataset S3: BAL 3157
Suppl. Dataset S4: BAL 4505
Suppl. Dataset S5: stool 9792
Suppl. Dataset S6: stool 9790
Suppl. Dataset S7: stool 1
Suppl. Dataset S8: CSF 7653
Suppl. Dataset S9: SRR533978
Suppl Dataset S10:SRR1553464
Suppl. Dataset S11: SRR1564804
Suppl. Dataset S12: su_38

Supplementary Table S1

ID	diagnostic entity	clinical symptoms	PCR Ct	pathogen detected conventional diagnostics
104	BAL	pneumonia	26	fluA, H1N1
3157	BAL	pneumonia	30	fluA, H3N2
4505	BAL	ARDS	29	*Chlamydophila Psittaci,*
9792	stool	AGE	21	Saporovirus
9790	stool	AGE	-	neg.*
1	stool	AGE	25<br>33	Norovirus GI,<br>Norovirus GII
7653	CSF	meningitis	32	Enterovirus B
7982	CSF	meningitis	33	Enterovirus B
7816	CSF	meningitis	33	Enterovirus B
7840	CSF	meningitis	33	Enterovirus B
7960	CSF	meningitis	32	Enterovirus B

Suppl. Table S1: PCR results obtained in conventional diagnostics for all clinical samples. *, sample was negative in PCRs testing for causative agent of acute gastroenteric infection (e.g. Caliciviruses; Rotavirus; Adenovirus). ARDS: acute respiratory distress syndrome; AGE: acute gastroenteritis.

Supplementary Table S2

contaminants environmental or handling of the samples	*GenBank accession number*
Retroviridae	
Avian myeloblastosis-associated virus	L10922.1
Avian myeloblastosis virus	S74099.1
Parvoviridae	
Parvovirus NIH-CQV	KM105951.1
unclassified circular DNA viruses	
Lake Sarah-associated circular virus-10	NC_029589.1
Lake Sarah-associated circular virus-28	NC_029617.1
Lake Sarah-associated circular virus-38	NC_029597.1
Lake Sarah-associated circular molecule 9	NC_029587.1
Lake Sarah-associated circular virus-19	NC_029609.1
Lake Sarah-associated circular virus-41	NC_029616.1
Avon-Heathcote Estuary associated circular virus 23	NC_026651.1
Dragonfly larvae associated circular virus-3	KP153450.1
Sewage-associated circular DNA virus-4	KJ547628
Sewage-associated circular DNA virus-15	NC_026257.1
Sewage-associated circular DNA virus-21	NC_026264.1
McMurdo Ice Shelf pond-associated circular DNA virus-1	NC_024477.1
McMurdo Ice Shelf pond-associated circular DNA virus-6	NC_024482.1
Microviridae (ssDNA)	
Gokushovirinae Fen672_31	NC_027642.1
Marine gokushovirus	NC_022790.1
Eel River basin pequenovirus	NC_026665.1
uncultured virus	
uncultured marine virus	186617*

Suppl. Table S2: Viral contaminants observed in UMERS; *: Tax ID

Supplementary Table S3

ID	reads	% host
A1	1,165,146	28.73
A2	1,402,943	84.99
A3	1,533,482	92.00
A4	1,577,814	46.68
A5	1,351,573	33.02
B1	4,921,044	03.32
B2	4,539,307	23.04
B3	2,765,321	10.93
B4	981,569	13.34
B5	3,928,682	25.59
B6	1,039,763	98.60
B7	1,033,802	94.24
B8	1,174,983	79.83
B9	3,802,217	77.92
B10	3,420,562	88.74
B11	10,363,612	47.13
B12	10,218,363	73.62
B13	9,684,714	97.92
B14	4,292,874	98.59
B15	11,309,016	60.11
B16	3,134,515	98.25
B17	3,026,905	65.78
B18	3,698,605	96.19
B19	1,108,907	67.85
B20	3,750,033	19.68
B21	4,879,963	90.37
B22	4,764,320	77.45

Suppl. Table S3: number of reads and
% host reads in samples included in the cluster analysis

Supplementary Figure S1

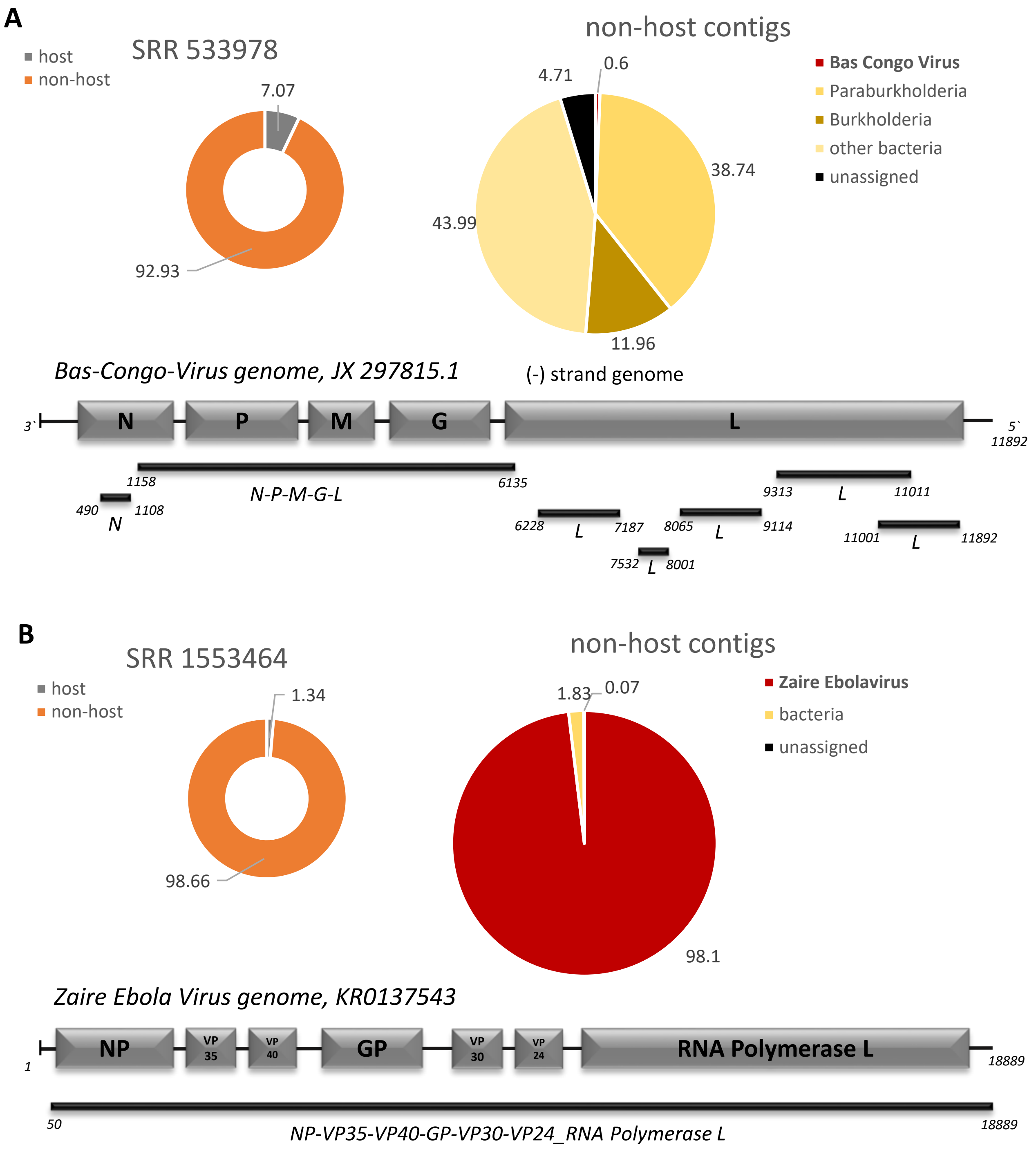

Supplementary Figure S1

C

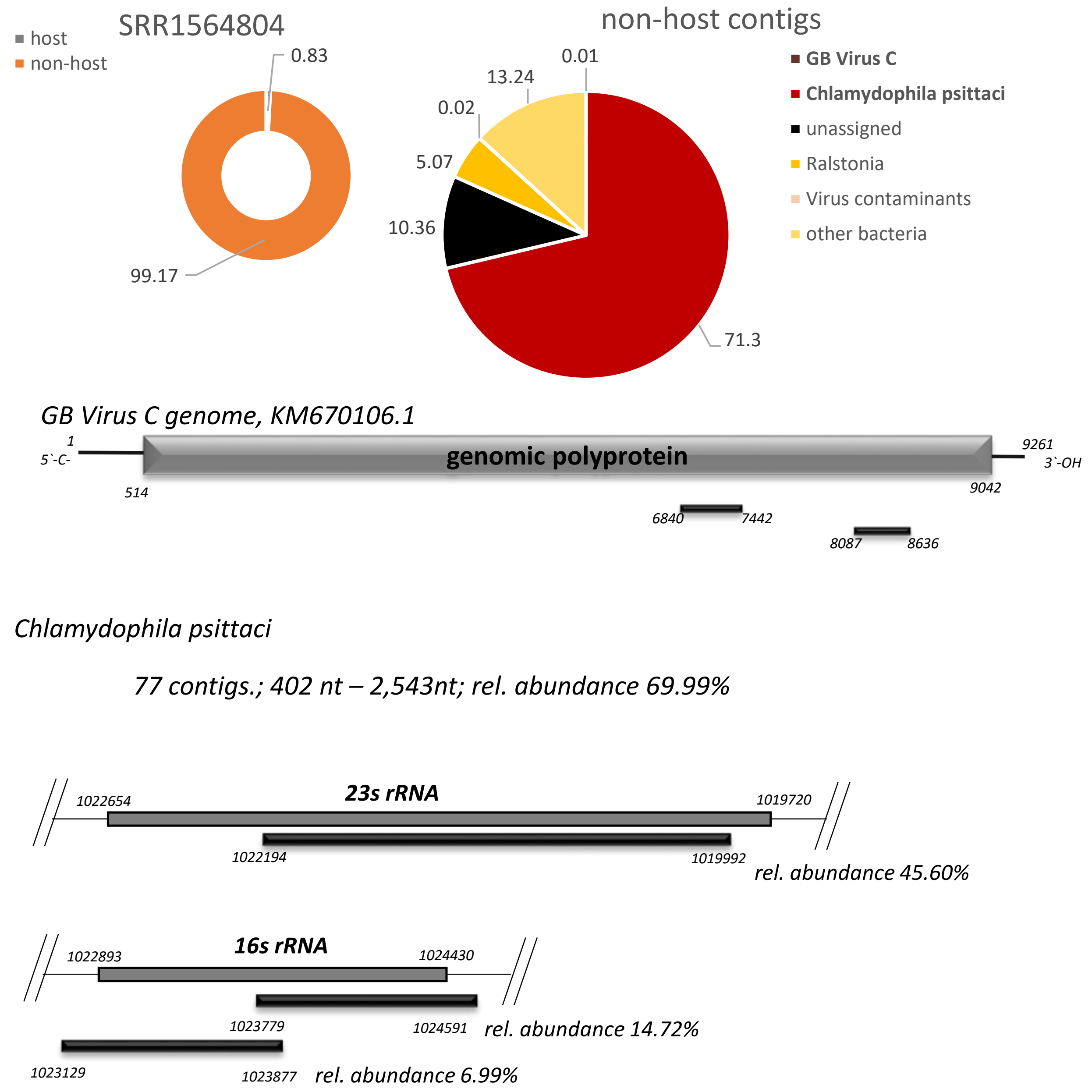

Supplementary Figure S2

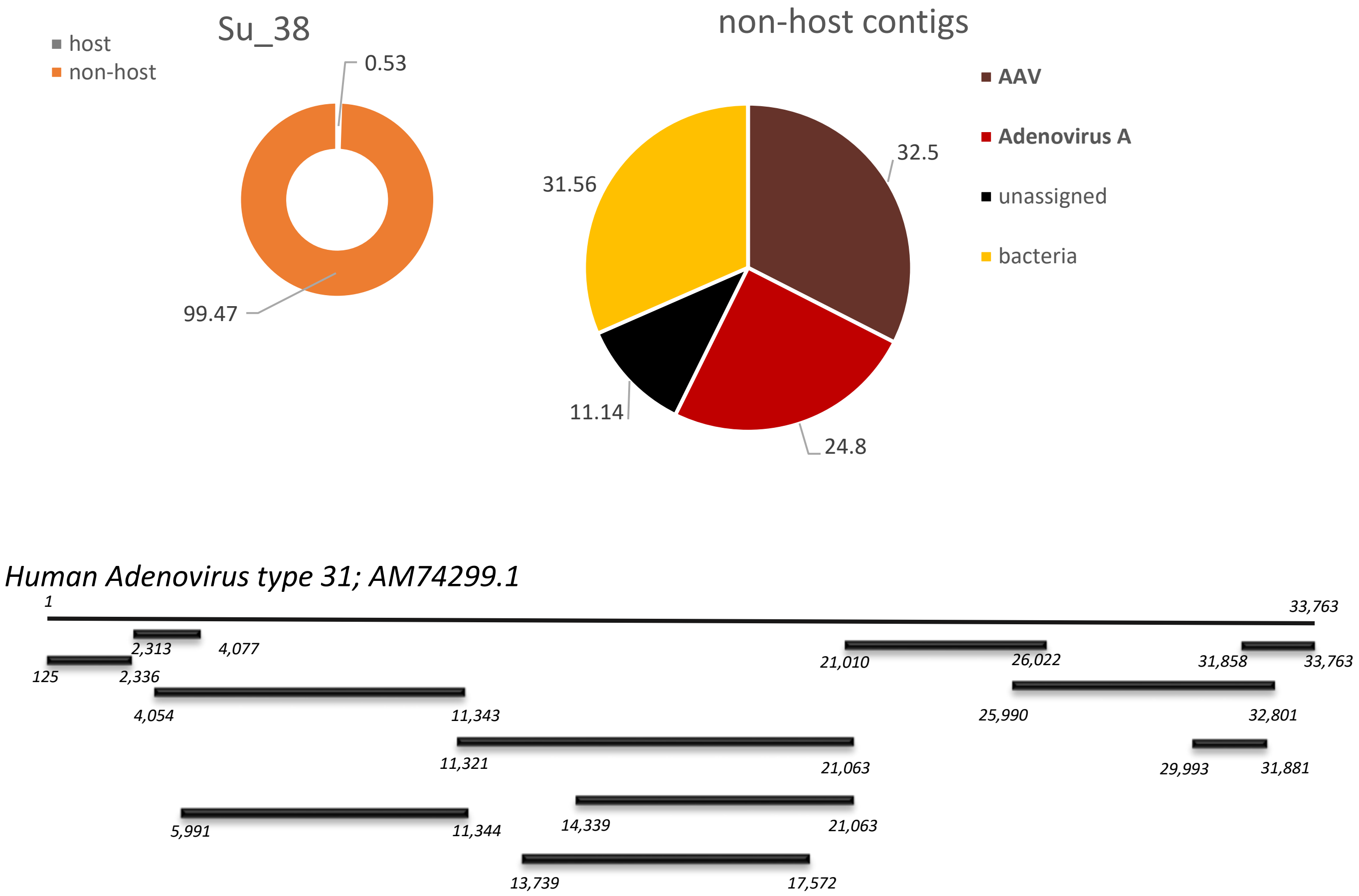

Supplement: Supplementary file 1 — supplementary material [file 41598_2019_52881_MOESM1_ESM.pdf]
